# Social justice and the medical librarian*

**DOI:** 10.5195/jmla.2019.712

**Published:** 2019-07-01

**Authors:** Elaine Russo Martin

**Affiliations:** Director of Library Services, Countway Library, Harvard Medical School, Boston, MA, elaine_martin@hms.harvard.edu

## Abstract

This lecture discusses social justice and the role that medical librarians can play in a democratic society. Social justice needs to be central to the mission of medical librarianship and a core value of the profession. Medical librarians must develop a new professional orientation: one that focuses on cultural awareness or cultural consciousness that goes beyond ourselves and our collections to that which focuses on the users of our libraries. We must develop a commitment to addressing the issues of societal, relevant health information. Using examples from medical education, this lecture makes the case for social justice librarianship. This lecture also presents a pathway for social justice medical librarianship, identifies fundamental roles and activities in these areas, and offers strategies for individual librarians, the Medical Library Association, and library schools for developing social justice education and outcomes. The lecture advocates for an understanding of and connection to social justice responsibilities for the medical library profession and ends with a call to go beyond understanding to action.

The lecture emphasizes the lack of diversity in our profession and the importance of diversity and inclusion for achieving social justice. The lecture presents specific examples from some medical libraries to extend the social justice mindset and to direct outreach, collections, archives, and special collection services to expose previously hidden voices. If medical librarians are to remain relevant in the future, we must act to address the lack of diversity in our profession and use our information resources, spaces, and expertise to solve the relevant societal issues of today.

## INTRODUCTION

I am truly honored to have been selected as the 2018 Janet Doe lecturer. Like the many Doe lecturers before me, being selected brings a host of mixed emotions, varying from amazement, joy, pleasure, and ultimately panic from the weight of the responsibility. The Janet Doe Lecture is an extensive examination of a topic related to health sciences librarianship. The topic is open ended, as long as the subject is under the very broad theme of philosophy or history.

Like most of the Doe lecturers before me, I too am not a philosopher nor a historian. My career as a medical librarian has been a journey both literally and figuratively. I have worked on both coasts and in the middle of the country, deliberately relocating to accept more advanced leadership positions. I have outreach experience in three Regional Medical Library network offices: the Pacific Northwest, the Greater Midwest Region, and the New England Region (in these last two, I led those programs). A defining moment in my career was the opportunity I had to work outside the United States, specifically in Liberia.

I started my library career as a library technician in a state medical society library and continued working in different support staff positions while attending library school part-time with the support of the Medical Library Association (MLA) Scholarship. My first professional job out of library school was as a reference/user education librarian, followed by administrative positions as a public services department head, an assistant director, then an associate director, and finally, a library director. The libraries in which I worked were in both private and public academic health sciences centers with a variety of health professional schools and affiliated hospitals. Because of this breadth of experience, the kinds of projects I have worked on, and the diversity of the communities in which I have worked, the topic for this lecture came naturally to me. I did not, like many previous Doe lecturers, experience a long and agonizing road for a search for my topic. For me, it was quite the opposite. In fact, the topic chose me and seemed a natural fit.

I fleetingly thought about and quickly dismissed discussing the work I have done and continue to do in promoting research data management as a fundamental new role for medical librarians or my project in public health information access and outreach, specifically my approach to providing real-time access to the literature for the public health workforce who have no affiliation with academic medical libraries. But I must confess my choice of topic comes from contemporary concerns about what is going on in our society today and is influenced by movements such as #BlackLivesMatter, #Metoo, #Enough, #Resistance, #NeverAgain, and #MarchforOurLives, and the proliferation of accepted terms such as “fake news” or “alternative facts.” When I started exploring social justice as my topic, I had some initial doubts as to whether or not I should go forward. I acknowledge that some librarians might feel uncomfortable with the topic and what is to follow. However, given what has gone on to threaten our country’s democratic values and principles, I am more convinced than ever that I had to discuss “Social Justice and the Medical Librarian.”

I think most of us would agree that democracy in any country depends on an informed electorate with equal access to quality information, knowledge, and education. Therefore, by definition, the library profession is an integral part of democracy. Specifically, what we medical librarians do—making evidence-based health information available to those who need it in order to help patients, families, and caregivers make better patient-care decisions—connects us to democratic principles and ideals of equal access to information and health care. As Louis Brandeis, a former Supreme Court associate justice and “militant crusader for social justice,” asked and answered, “What are American ideals? They are the development of the individual for his own good and the common good; the development of the individual through liberty; and the attainment of the common good though democracy and social justice” [1]. In the United States, we have the means for such democratic ideals, but we have not lived up to the potential [2]. American democracy is in danger because of an increasingly ill-informed public who are easily manipulated with sound bites and Twitter rants that lead to the infringement on the rights of the poor, the disenfranchised, the underserved, immigrants, people of color, women, and the lesbian, gay, bisexual, trans, and queer (LGBTQ) community.

With the continuing closures of hospital libraries and relentless budget cuts experienced by many academic medical libraries since 2008, coupled with the proliferation of many alternative options for accessing information, the need for medical librarians is being questioned, and our sheer numbers, ranks, and status are diminishing. If medical librarians are to survive as a distinct profession, then we must consider who we are as individuals, embrace our place as medical professionals in a democratic society, stand up for human rights and social justice, and assert our social responsibility.

Few Doe lecturers have touched upon the roles of medical librarians in a democratic society. Henry Lemkau Jr., FMLA, in his 2007 Doe lecture, said, “Our lives are informed and given purpose by the influences that surround us,” and he discussed our profession “in the context of the social and cultural worlds in which we function” [3]. I suggest that this discussion has never been more important than it is today, given the times in which we live (mass deportations, children separated from their families at the border, no sustained solution for the Dreamers, and a government shutdown over the border wall). I would add to the discussion the context of the users, not just the professions, whom we serve.

The importance of human values in medical librarianship was first highlighted by Martha Jane Zachert in her 1978 Doe lecture [4]. Previous to her, Doe lecturers defined the values of medical librarianship in terms of collection-building, self-image, organization and retrieval methods, and technological savvy. David Bishop’s definition of diversity, for example, only focused on specialization in the field of librarianship and did not touch on diversity with respect to populations [5]. But it was Zachert who wrote of developing new values for the medical library profession “related to man’s cognitive life and social, as well as to social and cultural institutions and the process of social change.” She identified professionalism as one of our most “enduring values...perhaps the keynote of our value system” [4]. In my view, professionalism goes beyond the tasks we do or what we call ourselves, but rather embodies who we are and what we stand for [5].

For the most part, Doe lecturers have shied away from discussing politics and its effect on their topics or the medical library profession. Estelle Brodman was an exception with a somewhat lengthy discussion of the effect of Hitler’s Nazis, the Vietnam War, racism, and the perils of the “pursuit of power” over the “pursuit of excellence” [6]. The 1989 Doe lecture by Rachael K. Anderson, AHIP, FMLA, was the first to explore the factors that have influenced who has been able to enter our profession, with a particular focus on racial and gender discrimination [7]. Gerald Oppenheimer, in 1988, was most adamant about our profession looking outward and “concentrating on values…which are directed outward to the society in which we live and work” [8]. He went on to describe the debate in MLA between those who felt we should leave human rights to others and those who felt the association should take a more active role in democratic ideals.

You may think given the title of my talk that it is also a political talk. Promoting equality and democratic ideals is not partisan. Rather, it is a talk about values and what Barack Obama named democracy with a small “d” [9]. It is a talk about social justice in our everyday work. It is a talk about how we can be relevant to the people we serve by providing information that improves everyone’s health, not just the privileged few. It is a talk about professionalism, and how we can incorporate and promote social justice into our profession, how we can become agents of societal change, and how we practice as medical librarians in the context of the times in which we live.

## DEFINING SOCIAL JUSTICE

—Of all the forms of inequality, injustice in health care is the most shocking and inhumane. Martin Luther King [10]

What do I mean then by social justice in the context of health care and medicine? While social justice and diversity are linked, social justice goes beyond representation. A term hard to define, social justice in medicine is:

the open acknowledgement of the dignity and autonomy of and delivery of high-quality medical care, to *all* members of society, regardless of gender, race, ethnicity, religion, sexual orientation, language, geography, origin, or socioeconomic background. [11]

It is the idea that health care employees work toward eliminating racial and ethnic disparities in health care [11].

The 2002 Institute of Medicine report, *Unequal Treatment*, first detailed a systematic examination of racial and ethnic disparities in health care. It clearly indicated that US racial and ethnic minorities were less likely to receive even routine medical procedures and more likely to experience a lower quality of health services, and called out the social responsibility of physicians toward their patients [12]. Social justice is not just an admirable idea. Social justice is a responsibility of the health care team to provide health services for every person, no matter where they live, what they do, or what lifestyles they lead. The case for social justice medical librarianship comes from our roots in the health professions and medical education. I hope this talk challenges our preconceived notions of the role librarians should play in social justice and suggests some specific actions.

There is a growing recognition amongst the health professions and medical educators that there is disparity in the health care delivered to ethnic, racial, gender, and other minority populations [13]. These disparities lead to patient dissatisfaction, noncompliance with treatment, and poorer health outcomes. Diseases such as tuberculosis and HIV are social diseases. There is recognition that the impact of societal and economic factors on the individual needs to be considered just as much as the bacteria of the disease itself [14]. There needs to be a broader awareness of the barriers to accessing health services among underrepresented groups. There also needs to be a comprehensive strategy in addressing them [12].

The Liaison Committee on Medical Education (LCME) standard 7.6 is one concrete step [15]. All medical schools in the United States and Canada must address the issues of social justice, cultural competency, diversity, and inclusion along with the need for medical students “to recognize and appropriately address gender and cultural biases in health care delivery” as part of the medical school accreditation process. Specifically, medical students must demonstrate an understanding of how culturally diverse perspectives of health and illness affect a person’s response to symptoms, disease, and treatment. The LCME standards also require students to understand how gender and cultural biases affect health care delivery [16]. Medical education is not alone: these themes are reflected in transforming the education of future nurses, public health workers, and other members of the health care team. If medical librarians are to maintain our status as members of the health professional team, then we, too, must take up the call. I would even go so far to say that future survival as a profession depends on it.

Rudolf Virchow, who is considered the father of social medicine, wrote in 1848, “Medicine is a social science, and politics nothing but medicine on a grand scale” [17]. Despite this, just like medical librarianship, the medical profession has been slow to respond to society’s issues. The medical professions’ late response to the HIV/AIDS pandemic and its effect on the LGBTQ community is only one example. Yet as evidenced by the LCME accreditation standards and the increased number of journal articles in *Academic Medicine* over the last decade, there seems to be a greater awareness among medical educators that teaching social justice to medical trainees and medical school faculty needs to be essential to educating future health professionals.

## SOCIAL JUSTICE AND MEDICAL SCHOOL CURRICULUM

There are examples of medical schools incorporating social justice into their curricular offerings:

- Through problem-based learning methods, medical students and faculty at the University of Hawaii have developed the Social Justice Curriculum Project, consisting of self-directed learning, action, and self-reflection [18].
- The University of California Davis Health System offers faculty development training on how to conduct interracial dialogue on race, racism, oppression, and privilege [19].
- In memory of Freddie Gray, faculty at Northeastern Ohio University have designed a curriculum to help trainees and faculty understand unequal access to health care and how physicians can work toward eliminating the injustices contributing to inappropriate care [20].
- The Human Rights and Social Justice Scholars Program at the Icahn School of Medicine at Mount Sinai is a preclinical training program in social medicine that incorporates service learning experiences with lectures, mentorship, research projects, policy and advocacy projects, and a seminar series [18, 21].
- The Harvard Medical School hosts the “Equity and Social Justice” series of lectures and dialogues focusing on history and context, culture and environment, health disparities, and leadership and skills development.

Other medical schools are going beyond the LCME guidelines and are beginning to also provide advocacy training to address social determinants of health. The LEADS Curriculum at the University of Colorado School of Medicine, the Scholarly Concentration in Advocacy and Activism at the Brown University Warren Alpert Medical School, and the Health Justice Scholar Track at Georgetown University School of Medicine are early examples that focus on empowering medical students to design and execute advocacy projects for social change.

I am fully aware of the criticisms of social justice and social justice education. Some may think it too politically liberal, or too politically correct, or too leftist or activist. Some may feel that they cannot express their conservative views in these settings. The goal of social justice is not to have us conform to one way of thinking but rather to give everyone the opportunity to be engaged, thoughtful community members who think critically about issues affecting the community. This is the tradition of a liberal education without having politically liberal connotations. It is where students, educators, and librarians recognize the complexity of the world and our interconnectedness. It is an opportunity for us all to reflect and think critically about these issues, develop tolerance for ambiguity, appreciate diversity, and respect the different views of others [22].

## THE CASE FOR SOCIAL JUSTICE AND THE MEDICAL LIBRARIAN

Like medicine, medical librarianship is not only an information science, but a human science. It is the search, retrieval, evaluation, and application of information to meet human needs to help health professionals, students, and patients make informed decisions about their health. Currently, much of what we do in medical libraries still focuses on developing vast libraries of print or online collections, enhancing informatics technology-based skills, and developing evidence-based best practices for delivering reference, education, or other services. While performing activities such as developing data science plans, digitizing special collections, and conducting systematic reviews are important, they need to be done in a social justice context.

Through a social justice lens, we would need to introduce more humanistic approaches to our work: refocusing our attention from serving ourselves and what is more efficient or effective for us to do in our libraries and shifting our focus outward. We need to focus on learning what our users want from us, learning how individual users experience the library, and tailoring our services and approach to their individual needs and experience. Social justice librarianship involves developing a personal and professional approach in which the practice of medical librarianship puts the user’s interests and needs front and center. Like medicine, we have been slow to recognize what our social responsibility to those we serve is and that there is no one size fits all to the services we provide.

This lecture critically discusses this concept of social justice and proposes that medical librarians must go beyond the traditional approaches to thinking about their work and must develop a deeper understanding of and connection with the social responsibilities of the health professions and people we serve. Medical librarians must develop a new professional orientation—one that fosters a critical awareness or critical consciousness of going beyond the self to others and a commitment to addressing the issues of societal relevant health information. This new professional orientation or identity places information science in a social and cultural context. It is coupled with a recognition of societal injustices with respect to access to health care and health information and a search for appropriate action.

I will suggest a framework for medical librarianship social justice, identify the fundamental roles and activities in these areas, and suggest strategies for individual librarians as well as MLA, the Association of Academic Health Sciences Libraries (AAHSL), and schools of library and information sciences (LIS) for developing education and outcomes. It is my hope that, through this lecture, you will gain an understanding of and connection to the social responsibilities of medical librarianship, develop an individual approach to reflective professional practice, and be inspired to action.

## THE SOCIAL JUSTICE EDUCATION DEBATE

While there is more agreement today that health professionals need to be trained in cultural competence, multiculturalism, and social justice, there is a debate regarding the curriculum and the accompanying teaching methods. In academic medicine, proponents of critical consciousness theory view it as a way to help refocus the current methods of health professional education. Medical education focuses on developing procedurally competent physicians. Adding social justice to health education brings physician training back to its original mission of developing socially conscious health providers who focus on the patient-doctor relationship and to “inform an appropriate crucial pedagogy for fostering compassionate, humanistic, socially conscious health professionals who act as agents of change” [23].

The early efforts in social justice medical education curricula stress developing competencies in multiculturalism. These efforts use the categorical approach where attitudes, beliefs, and behaviors of specific cultures and groups are defined, outlined, taught, and memorized. While acquiring multicultural competence implies learning about multiple and diverse cultures, it is limiting. It often leads to oversimplifying and stereotyping certain groups. This is an unintended outcome. This realization has led to new teaching methodologies that focus on developing a set of skills to assess individually what factors might affect a patient’s care or developing critical consciousness [11]. Medical educator Delese Wear cautions that it is not enough to be culturally competent and, in fact, multicultural competency–based education can become “a medical education paradigm in which the notion of ‘novelty’ replaces that of ‘equality’ in approaches to treating patients” [20]. Kumagai argues that a competency-based approach to social justice education is not appropriate, as linear modes of learning may help the student accumulate knowledge but not necessarily understanding. He argues for new ways of thinking about how students learn or know. Teaching social justice to support professionalism calls for new content and new teaching methods [24].

The concept of the medical professional who practices actively in the world involves a paradigm shift away from passive learning of new knowledge and skills to more active learning. It requires developing a new professional identity with a reflective orientation to the understanding of self, professional self, others, and the world. It has its roots in “Critical Conscious Theory,” a term proposed by Brazilian activist and theorist Paulo Freire in his 1970 work, *Pedagogy of the Oppressed*. Critical consciousness goes beyond critical thinking. It is the “ability to intervene in reality in order to change it.”

The link from education to democracy is implicit in Freire’s work. Learners act as subjects in the creation of democracy in education through telling and listening to experiences. Consciousness is a sense of one’s personal and collective identity, an in-depth understanding of the world, and a penchant for action against oppression [25]. Friere saw the act of dialogue as an act of proclaimed equality: dialogue, curiosity, creativity, and critical consciousness actively seek to intervene and change society. He observed the educational system in Brazil, which focused on spoon-feeding or banking and depositing knowledge to the masses, as oppressive. He advocated for more active learning and questioning as a way of freeing the individual. He felt that learners needed to connect to their own personal, cognitive, and emotional experience; to engage with others through dialogue; and to emancipate themselves and others through praxis (applying theory to action). This capacity to connect with one’s position in society and engage in dialogue about inequities depends upon critical consciousness—a reflective reading of the world. From consciousness, learners could act as agents of change. Social justice education is actively learning by doing.

Friere’s theory of critical consciousness has been applied to educational change in various disciplines, most recently, medical education:

A Friere critical teacher is a problem-poser who asks thought provoking questions and encourages students to ask their own questions…students experience education as something they do, not as something done to them…educators…are challenged to de-privilege their own power and authority and become informed, experienced and knowledgeable facilitators of student learning rather than depositors of information into the mental vaults of learners. [26]

Students are both teachers and learners and vice versa.

Kumagai expands on Friere’s work as applied to teaching social justice in a medical context. He calls on the teachings of German philosopher Jürgen Habermas and his framework for knowing and communicating. Teaching social justice requires another type of learning, by which individuals (and, indeed, groups and societies as a whole) formulate new ways of understanding reality, of interacting with others, and of perceiving their own identities. Habermas identifies three primary ways in which we generate knowledge. The first is “Work” or instrumental action—knowledge based upon empirical investigation and governed by technical rules. Scientific research is an example of this domain. In librarianship, this may equate to technical work in maintaining systems and collections. The second is “Practical Knowledge.” This domain identifies human social interaction or “Communicative Action.” It is defined by “building consensus or standards” in order to determine appropriate action. Legal and social sciences belong to the “Practical.” In medical librarianship, “Practical” is the way we help develop clinical practice. The last is “Emancipatory,” or “self-knowledge” or “Self-reflection.” This involves:

interest in the way one’s history and biography has expressed itself in how one sees oneself, one’s roles and social expectations…Insights gained through critical self-awareness are emancipatory. Knowledge is gained by self-emancipation through reflection leading to a transformed consciousness or changed perspective. [27, 28]

For medical librarians, this can include approaching the reference interview from the user’s perspective and having a greater awareness of our own biases prior to providing canned searches or information packets. The medical librarian needs to have empathy for the individual asking the reference question and treat every question as an individual need. Habermas’s framework of knowing is important to consider as we think about a social justice framework for medical librarianship.

Although medical training, appropriately so, must focus on developing technical skills and consensus-driven understanding of best clinical practice, not all education to prepare health professionals to work in society arises from these ways of knowing. Implicit in the efforts to introduce more humanistic care into medicine and to address topics as social justice and human values into professional training is a requirement that a new way of knowing does not involve just knowing facts and figures. It involves the personal and professional orientation toward self and others—a way of being in the world—in which the practice of medicine has the patient’s interests at the forefront.

This way of being cannot be taught through traditional lecture or classroom settings but must be acquired through reflection, dialogue, and experience [28]. This type of education engages learners and teachers in the exploration of self and others through the use of narratives, reflective writings, comics, art, theater, and film. The emphasis is to engage in reflective interaction with a play, film, or essay; with a patient; and with each other in opportunities to grapple with moments of uncertainty and discomfort, and to go ultimately beyond discussion to action in the world. Medical libraries, schools of LIS, and our medical library associations need to emulate the efforts from our health professional colleagues when it comes to social justice education. We need to introduce more of these ways of learning and concepts into our formal and informal curriculum for medical librarians.

## DIVERSITY IN LIBRARIANSHIP

Let me now turn to diversity in our profession. Though not the same as social justice, diversity is important for achieving it. While the library community considers diversity to be a core value, the library sector has fallen short, despite ongoing initiatives that focus on the recruiting minority librarians.

What do we mean when we say diversity, and why does diversity matter? Diversity matters because we want our libraries to be reflective of the diverse communities in which we work and the users we serve. A recent McKinsey analysis of 366 global public companies found that those companies with greater ethnic diversity are 35% more likely to outperform their peers: “Diversity matters because it is an opportunity to be innovative, to leverage gifts and talents of all our people” [29].

For at least the last two decades, many academic libraries have established diversity committees, residency programs, and fellowship opportunities for minorities in order to increase representation in the workforce. In recent years, some academic librarians have worked toward understanding this issue and have spoken out about the shortcomings of efforts to diversify the profession and advocate for social justice [30]. Professional associations have grappled with the issue of diversity and initiated efforts to increase the representative numbers of library employees of color in librarianship. Library organizations have implemented programs toward effecting change in the racial and ethnic makeup of the profession. In 1997, the American Library Association (ALA) began the Spectrum Scholarship Program meant to address ethnic under-representation in the library community. More recently, the Association of Research Libraries began providing a number of diversity initiatives surrounding recruitment and career development of underrepresented ethnic groups, including the “Initiative to Recruit a Diverse Workforce” and the “Leadership and Early Career Development Program,” among others. The Association of College and Research Libraries has in recent years formed a Diversity Alliance. MLA has had a Scholarship for Minority Students and recently formed the Diversity and Inclusion Task Force; AAHSL has acted similarly.

Despite these efforts, librarianship remains a primarily white female profession. The Ithaca S+R report, funded by the Andrew Mellon Foundation, called the “Inclusion, Diversity, and Equity Survey,” is the most recent attempt to measure representational diversity in libraries and documents the lack of racial and ethnic diversity in the profession. It found that over 75% of employees at academic research libraries were white. And as positions become increasingly senior, they also become increasingly white [31]. The report also found that library leaders or administrators are 89% white and non-Hispanic. It also noted that many non-white staff members work in roles such as technical services, processing, and so on, which are being phased out of libraries as they move from print to electronic collections. One of the study’s coauthors Roger Schonfeld wonders whether “There is in fact a risk that libraries will become not more diverse in the future but potentially less diverse in the future if action isn’t taken” [31]. The report gives credence to minority staff who have faced barriers to advancement.

These issues become even more pronounced as the national population has grown markedly more diverse. The US population (2013 figures) is 62% white, with a projection for 2060 that white people will make up only 40% of the population [32]. Using the ALA Diversity Counts data and comparing it to the US Census data for 2013 and US Census projections for 2060, it is clear that we are far from reflecting our country’s diversity.

However, diversity has a much broader definition than race and ethnic representation and encompasses combatting discrimination based on age, sex, sexual orientation, gender identity, gender expression, religious background, language, or disability. The ALA Diversity Counts data go beyond highlighting the disparity for race and ethnicity. We lack other forms of diversity in libraries as well, though demographic data for areas other than race and ethnicity are less well defined or tracked. The Diversity Counts authors highlight the low employment of librarians with disabilities, given the increase between 1990 and 2000 of people self-identifying as having 1 or more disabilities. While 19% of 21–64 year olds self-identified as having a disability on the 2000 US Census, the percentage of credentialed librarians was 4% [32]. The recent Association of American Medical Colleges (AAMC) report called “Learners and Disabilities” is similarly discouraging. Only 2.5% of 1,500 medical students self-identified as having disabilities and needing accommodations [33]. The report highlights the barriers that medical students with disabilities experience in their training.

Going back to librarians, the Diversity Counts report states, “Credentialed librarians are predominately women, ages 45–54, and white. They are not limited by disability and work full-time” [32, 34]. This lack of diversity in libraries in regard to race, ethnicity, disability, and other factors distance the very communities we seek to serve. And it suggests a proportionally less diverse library workforce on the horizon [34].

We need to ask ourselves why diversity does not happen despite libraries making diversity a priority. Why are these efforts not making any meaningful difference? Why are we bringing people from underrepresented groups into our libraries at the same rate they are leaving the profession [34]? What are the barriers to diversifying employees? When asked this question in the Ithaka survey, library directors recognize there is a problem but identify the problem as something they cannot control. They blame external factors, such as lack of a talent pool or geographical location, rather than internal factors such as unconscious bias in the interview process. [31]. In addition, they identify their libraries as more inclusive than the library community as a whole, whether or not that is the case [31]. Yet, we know that in comparing the percentage of racial and ethnic subgroups in the US population, the distribution in library assistant positions is more or less proportional, whereas the distribution in professional librarian positions is not, the latter skewing heavily to an overrepresentation of white people. Diversity Counts confirms this discrepancy [35].

One strategy may be to look internally for higher education and promotional opportunities for library assistants in our own libraries. Perhaps looking in our libraries and not outside the environment is where the problem really lies. When I was at the University of Illinois at Chicago, for example, the university librarian, Sharon Hogan, sponsored a program for underrepresented minority library assistants to attend the master’s of library and information science (MLIS) program at the University of Illinois Urbana-Champaign. Selected employees went to school on weekends (tuition free), the library provided transportation, and upon graduation, students/employees were guaranteed a paid professional position in the library system. It should be easier today to implement programs like this, especially since there are so many online LIS degree programs available. Eligible employees could be given educational leave to complete coursework, allowed to use work computers, and receive tuition benefits.

Common library hiring processes and practices also contribute to the lack of diversity. We need to examine the criteria for job descriptions, recruitment processes, and hiring practices to open up the pipeline for underrepresented groups to enter the profession and provide opportunities for advancement. While we discuss and say we encourage the recruitment of library workers from diverse backgrounds, our application requirements are not designed to celebrate the experiences of diverse applicants. We need to reframe application requirements, materials, and interview questions in ways that make sense for a variety of applicants’ experiences [36].

## SOCIAL JUSTICE IN MEDICAL LIBRARIANSHIP

In earlier portions of this talk, I discussed how medical educators and health professional students have incorporated social justice, through critical consciousness, into their work and studies. I have also discussed how our profession has been challenged in achieving success in diversity despite our efforts. Though social justice has not been the focal point for the medical library profession, there are examples in which individual medical librarians have been active in what I would call social justice outreach.

One such example is the response of some of our members to the appearance of HIV/AIDS in the early 1980s, a disease that was social and stigmatized the populations it affected. While only a handful of articles about this new disease appeared in peer-reviewed journals in the early 1980s and the disease was never recognized until after the Ronald Reagan presidency, new and different sources of information were developing to fill the void. Gay and community-based organizations primarily in New York and San Francisco, where effects of the disease were most felt, were collecting and distributing information about treatment and prevention; often with the help of medical librarians working with these organizations. The first MLA presentation on this topic, “The AIDS Information Crisis: Confluence of the Roles of Information Creator, Seeker, and Provider,” by David Ginn, which was delivered at the 1987 annual meeting in Portland, Oregon, chronicled the information phenomenon in the gay and community-based organizations and called for the AIDS information gap to be addressed by our profession [37].

At the same time, M. Kent Mayfield, MLA headquarters continuing education program leader, enlisted Ginn and Richard Stevens, AIDS director of the Health Council of South Florida, to develop an AIDS information course outlining the new and expanding types of information that existed in the community setting. In the meantime, Gerald J. Perry, AHIP, FMLA, and Jeffrey T. Huber were publishing important books on AIDS information resources and terminology. Perry later went on to form the LGBT Health Sciences Librarians Special Interest Group (SIG) in MLA. In 1994, the National Library of Medicine (NLM) developed a special funding mechanism for HIV/AIDS community-based information access, in which some of us have partnered over the last twenty plus years, bringing information retrieval, skills development, computer acquisition, and Internet access to this affected community.

There are numerous other projects funded by NLM in which medical librarians partnered with community-based organizations to enhance the health information literacy of underserved populations. As they are too numerous to name them all, I will highlight a few I am most familiar with. Projects included partnerships with mental health patients and providers. E-mental health programs, coordinated through the Countway Library with partners at the Massachusetts Department of Mental Health and the Dartmouth College and Harvard University Medical Schools, were designed to enhance the health information literacy of seriously ill mental health patients and their families, focusing on co-occurring disease. Persons with serious mental illness have high mortality rates, tend be smokers, experience obesity, and present with multiple physical health concerns. This project focused on information related to physical as well as mental health resources. Another project I worked on while in Worcester addressed the opioid crisis. We worked with the city’s first alternative high school for addicted teens. Medical librarians worked with students, their parents, and their teachers to create a resource room with Internet, computer, and pamphlet materials and sponsored speakers who focused on options for recovery, addiction counseling, and the decriminalization of addiction.

When NLM broadened its attention to serving consumers in addition to health professionals, its funding for health information outreach to minorities, underserved, and vulnerable populations through the National Network of Libraries of Medicine (NNLM) has been and continues to be a critical vehicle for social justice librarianship. Medical librarians partnered with public libraries to provide consumer health collections and access to MedlinePlus, computers, and Internet connections as well as training for public librarians. In 2002, for example, medical librarians from the Yale University Harvey Cushing/John Hay Whitney Medical Library partnered with the New Haven Free Public Library to develop a Consumer Health Information Center with collection and information services for the city’s public library users who are traditionally from minority and economically disadvantaged groups. Then Library Director Regina Kenny Marone received a recognition award from the city for her leadership in this project.

Another example is the SPIRAL project from Tufts University Hirsch Health Sciences Library. SPIRAL stands for “Selected Patient Information Resources in Asian Languages.” Partnering with the Chinatown community in Boston, Tufts University health sciences librarians and community health center providers developed a free online website with patient health information resources in languages such as Chinese, Vietnamese, Cambodian, Korean, Thai, and Japanese.

Medical librarians working in international librarianship in conjunction with health care providers to expand medical education curricular and accompanying library resources in countries such as Liberia speak to our profession’s commitment to improving health through equal access to information in a global society. The societal impacts of diseases such as Ebola on a country like Liberia are major, threatening the peace process, causing psychological distress, and resulting in numerous losses of life for the citizenry and health care providers, causing a breakdown of the health care and educational systems. Cultural considerations such as burial practices and wide distrust of traditional healers as well as a lack of basic infrastructure (regular electricity, limited Internet access) hindered efforts in the beginning to control the disease. In a country like Liberia, which was recovering from decades of civil war and whose people experienced years where educational institutions could not operate, basic library services such as print textbooks, a manual check-out system, and copy cataloging served the medical school faculty and students well. The Ebola outbreak resulted in the closing of the medical school, but post-outbreak that basic library was continuing to function when the medical school reopened, and luckily, all the staff we worked with survived the crises, though some of the students had died.

The Countway Library of Medicine, Harvard Medical School, is extending the social justice mindset beyond direct outreach services to incorporating a public history perspective to its medical archives and special collections. The project uses a pubic history perspective to apply history to real-world problems. The project, “Equal Access: Stories of Diversity and Inclusion at Harvard Medical School,” focuses on collecting resources that fill in the gaps in the history of medical education. The project includes oral histories and papers from students and faculty who are underrepresented in medicine, including the first women and African American students and faculty. The intention is to use these collections to create a “dialogical” with collaboration and input from the community whose collections are being archived and personal stories recorded in order to more fully interpret and understand the issues surrounding barriers to accessing medical education and why this history is important.

The success of these and similar projects depends not on just going into the community and making resources available. All these outreach projects involve getting to know the community and its cultures, developing an equal partnership with the community, showing respect for cultural norms and practices, and then tailoring services to these.

Medical librarians’ obligation in providing consumer health information services to the public is a relatively recent phenomenon. MLA’s recognition of the Consumer and Patient Health Information Section, the subsequent Consumer Health Information Specialization, and most recent announcement of nominations for the newly endowed MLA Consumer Health Librarian of the Year Award have cemented what was once regarded as a controversial new role into accepted practice.

## CALL TO ACTION

—A man dies when he refuses to stand up for that which is right. A man dies when he refuses to stand up for justice. A man dies when he refuses to take a stand for that which is true. Martin Luther King [38]

If medical librarians are to remain relevant in the future, I believe we must deal with the lack of diversity in our profession, actively create our future using humanistic approaches, and use our information resources, spaces, and expertise to solve relevant issues of today. These include addressing the social and racial injustices in health care. We can use our library spaces to encourage critical consciousness conversations about the hard issues such as racism, sexism, gun violence, health disparities, climate change, and other societal issues affecting the health of the nation.

Our libraries can be the community centers where these conversations, open dialogue, and civil debate should be encouraged and can occur. In addition, we can provide the quality information and data to help participants in these conversations critically evaluate and sift out the fake news from the real news. It is our responsibility to provide access to accurate, trusted information as well as the tools and skills to critically evaluate that information in ways that promote diversity, equity, inclusion, and social justice. In addition, we need to collect, preserve, and make available information by, for, and about marginalized communities and fill in the gaps in the history of medical education. In 1962, British librarian Douglas John Foskett wrote *The Creed of a Librarian: No Politics, No Religion, No Morals*, in which he argued “the librarian ought virtually vanish as an individual person, except in so far as his personality sheds light on the working of the library” [39].

I disagree with Foskett. In my opinion, the notion of library neutrality is a myth. Our buying decisions, resources, budgets, and even locations are not neutral. We contribute to certain kinds of inequalities through our acquisitions decisions, our cataloging practices, our classification schemes, our controlled vocabularies, our use of commercial search engines, and even where we are located in our hospitals or academic campuses. We regularly practice self-censorship in book selection by primarily buying from corporate vendors. Neutrality is “a code word for the existing system. It has nothing to do with anything but agreeing to what is and will always be…Neutrality is just following the crowd” [40]. A neutral stance does not care about human rights violations. Neutrality does not account for social and economic inequalities. The notion that the medical library is a social institution that serves as a community center for its users is not neutral. Medical librarians cannot be neutral and be trusted advocates for their communities, especially the underserved. I believe that medical librarians can be forces for social good. This is crucial to our future and to the health of our local communities and a sustainable global community.

What can medical librarians do? We need to move beyond awareness to action. We must address the whiteness of our profession and develop real strategies for doing so [36]. We must acknowledge that our libraries have never been and are not neutral. We must bring our libraries closer to the communities we serve. We need to confront the societal responsibilities of LIS and develop curricula that emphasize preparing future librarians for leadership roles in activism, community service and engagement, rights, and justice. We need to shift our focus to the people and communities we serve and de-emphasize collections. Medical librarians in partnership with medical educators can and should provide students with opportunities, resources, and spaces that encourage open dialogue on social issues, broaden their horizons, encourage empathy for patients, and use these experiences to make a difference. Medical librarians should embrace a humanistic approach to professional practice, one that embraces the whole person, not just the physician scientist, and supports curricular efforts that expand the humanities in medicine. We need to offer services, programs, and resources that reflect the diversity of the communities we serve and provide outreach to and advocacy for and with underserved communities in our local communities. We need to support open access publishing and advocate for policies that increase access to information for all [30].

Medical librarians, our professional associations, and LIS schools can take specific actions to promote social justice. We can offer open dialogues and discussions on social justice topics at our library conferences. We can develop hiring practices that recognize a variety of experiences, application formats, and interview styles to encourage diverse applicants to apply. We can commit to opening the pipeline into medical librarianship through innovative pathways for library assistants to attain the MLIS. We can make concerted efforts to hire staff from diverse backgrounds that reflect US census data. We must develop a welcoming and inclusive environment for all employees and library users in our workplaces. We must welcome all comers into our library conferences. We must develop an approach to professional practice that considers user needs and experiences first and offer library services, resources, and programs that reflect the needs of our users, especially those who may be marginalized. We can adopt a public history perspective to building special collections and archives, filling in the gaps. We can adopt and enforce practices and policies that show respect for *ALL* library users, even when these may be inconvenient for us. We must implement outreach and advocacy programs in equal partnership with underserved populations, respecting cultural norms, in order to increase access to health information throughout the world. In the LIS schools or programs, we need to add courses into the curriculum that focus on diversity and social justice topics; and we need to increase the number of LIS faculty from diverse backgrounds.

## CONCLUSION

—Injustice everywhere is a threat to justice everywhere. Martin Luther King [41]

Librarian of Congress Archibald MacLeish said, “Librarians must become active not passive agents of the democratic process” [42]. Social justice and social responsibility are core values of the medical profession. Medical librarians have been an untapped resource for this important work. Diversity, inclusion, and social justice are the defining issues for the present and future of the medical library profession. Positive outcomes from this work will be cementing the relevance of the medical librarian as a member of the health care team. Medical librarians who practice their profession through a social justice lens have the potential to have a meaningful impact on transforming the health of the public, especially the marginalized.

Medical librarians are no strangers to change and evolving roles. We have learned new technologies and adapted them into our library workflows. We have moved from a focus on print to electronic resources. We have added online searching, expert searching, systematic reviews, and in-person and online teaching to our portfolios. We work outside the library as informationists and are embedded into research or clinical teams. We have learned how to write data management plans and teach researchers and graduate students how to use electronic lab notebooks. Social justice medical librarianship is one more change in the evolution of our profession. We can do this. We must do this. The future of our profession depends on it.
