# Correction

**DOI:** 10.5195/jmla.2019.814

**Published:** 2019-10-01

**VOLUME 107**

**107(3) July, page 302**

Martin ER. Social justice and the medical librarian. J Med Libr Assoc. 2019 Jul;107(3):291–303. DOI: http://dx.doi.org/10.5195/jmla.2019.712.

Page 302: The quote by Martin Luther King Jr. should be:

—Injustice anywhere is a threat to justice everywhere. Martin Luther King [41]

